# Impacto da Ablação por Cateter na FEVE em Pacientes com Fibrilação Atrial e Insuficiência Cardíaca com Fração de Ejeção Reduzida: Uma Revisão Sistemática

**DOI:** 10.36660/abc.20260117

**Published:** 2026-06-26

**Authors:** Antonio Maria Zacarias Araújo Monteiro, Maria Giovanna da Cruz Tocantins, Jéssica Batista Souza, Renan de Jesus da Silva Albuquerque, João Vitor Ferreira de Oliveira, Roni Oliveira Pinheiro, Ster Marques de Lima Silva

**Affiliations:** 1 Centro Universitário do Estado do Pará Belém PA Brasil Centro Universitário do Estado do Pará, Belém, PA – Brasil; 2 Universidade do Estado do Pará Belém PA Brasil Universidade do Estado do Pará, Belém, PA – Brasil; 3 Universidade Federal do Pará Belém PA Brasil Universidade Federal do Pará, Belém, PA – Brasil

**Keywords:** Insuficiência Cardíaca, Fibrilação Atrial, Ablação por Cateter, Tratamento Farmacológico

## Abstract

A fibrilação atrial (FA) é uma condição que frequentemente acomete pacientes com insuficiência cardíaca com fração de ejeção reduzida (ICFEr), estando associada à piora da fração de ejeção do ventrículo esquerdo (FEVE) e a desfechos clínicos desfavoráveis. A ablação por cateter tem sido proposta como estratégia para restaurar o ritmo sinusal; entretanto, seus efeitos sobre a FEVE permanecem heterogêneos na literatura. Avaliar o impacto da ablação por cateter, em comparação à terapia medicamentosa, sobre a FEVE em pacientes com FA e ICFEr. Revisão sistemática de ensaios clínicos randomizados conduzida de acordo com as diretrizes PRISMA. O desfecho primário foi a melhora da FEVE, enquanto os desfechos secundários incluíram mortalidade cardiovascular, mortalidade por todas as causas e hospitalizações por IC. Outros desfechos exploratórios também foram considerados. Os ensaios clínicos incluídos demonstraram melhora da FEVE em pacientes submetidos à ablação por cateter quando comparados à terapia medicamentosa. No estudo CAMERA-MRI, foi observado aumento absoluto de 18,3% na FEVE após seis meses. No estudo CASTLE-AF, a ablação também se associou a menor ocorrência de mortalidade cardiovascular e hospitalizações por IC. Contudo, devido à ausência de metanálise, não foi realizada síntese quantitativa dos efeitos. Os estudos sugerem que a ablação por cateter pode estar associada à melhora da FEVE e a desfechos clínicos mais favoráveis em pacientes com FA e ICFEr. Entretanto, devido à ausência de síntese quantitativa, esses achados devem ser interpretados com cautela.

## Graphical abstract

**Figura Central:**
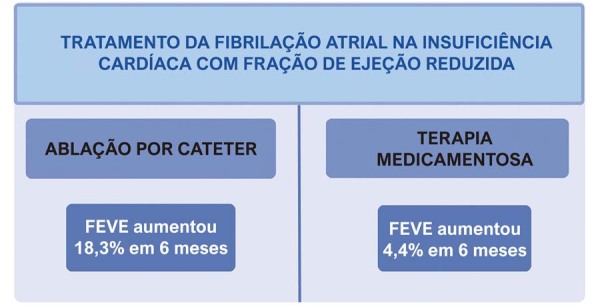
Impacto da Ablação por Cateter na FEVE em Pacientes com Fibrilação Atrial e Insuficiência Cardíaca com Fração de Ejeção Reduzida: Uma Revisão Sistemática

## Introdução

A insuficiência cardíaca (IC) é uma síndrome decorrente de alterações no enchimento e/ou na ejeção ventricular, associada a elevada mortalidade e significativa carga de complicações clínicas, dentre elas dispneia, limitação ao esforço e edema de membros inferiores.^1^ Tradicionalmente, é classificada segundo a fração de ejeção do ventrículo esquerdo (FEVE) em três categorias: preservada, intermediária e reduzida (ICFEr, FEVE ≤ 40%),^1^ sendo esta última responsável por cerca de 50% das internações por IC.^2^

A fibrilação atrial (FA) é frequente nesse contexto, acometendo cerca de um terço dos pacientes com insuficiência cardíaca com fração de ejeção reduzida (ICFEr).^3^ A coexistência dessas condições associa-se a pior prognóstico, com maior risco de acidente vascular cerebral, hospitalizações e mortalidade.^4,5^

O manejo terapêutico da FA na ICFEr tradicionalmente prioriza o controle da frequência ou do ritmo sinusal. Os betabloqueadores constituem a primeira escolha na terapia de controle da frequência cardíaca.^6^ Contudo, seu uso permanece controverso, uma vez que não se associou a reduções significativas de mortalidade em alguns estudos.^7,8^ O controle do ritmo por antiarrítmicos, principalmente a amiodarona, constitui outra alternativa terapêutica de suma importância; entretanto, apresenta limitações devido à ocorrência de eventos adversos relevantes nessa população.^9,10^

Nesse cenário, a ablação por cateter emerge como alternativa terapêutica ao uso de antiarrítmicos, sendo associada à melhora da FEVE, além da redução de desfechos clínicos relevantes, como mortalidade e hospitalizações por IC.^1 1,1 2^ Contudo, observa-se escassez de estudos que avaliem exclusivamente os efeitos da ablação em pacientes com ICFEr, sobretudo em relação ao desfecho FEVE. Além disso, há considerável heterogeneidade entre os estudos quanto aos critérios de inclusão, protocolos de ablação, terapias medicamentosas e definição de sucesso do procedimento, o que limita a generalização e a interpretação dos resultados.^13,14^

Diante desse contexto, esta revisão tem como objetivo avaliar a eficácia da ablação por cateter em comparação à terapia medicamentosa na melhora da FEVE em pacientes com FA e ICFEr, considerando a heterogeneidade dos estudos disponíveis.

## Métodos

Trata-se de uma revisão sistemática conduzida conforme as diretrizes do *Preferred Reporting Items for Systematic Reviews and Meta-Analyses* (PRISMA),^15^ com protocolo previamente registrado no PROSPERO (CRD420251044229).

### Busca e seleção bibliográfica

A pergunta norteadora foi formulada com base na estratégia PICO, considerando pacientes com FA e ICFEr, comparando a ablação por cateter à terapia medicamentosa quanto à melhora da FEVE.

A busca foi realizada em março de 2025 nas bases PubMed, BVS, Cochrane Library, SciELO e EMBASE, utilizando os descritores “*Atrial Fibrillation*”, “*Heart Failure*”, “*Catheter Ablation*” e “*Drug Therapy*” (DeCS/MeSH), combinados com o operador booleano AND. Os estudos identificados foram importados para a plataforma Rayyan^®^ e avaliados independentemente por dois revisores.

### Critérios de inclusão e exclusão

Foram incluídos ensaios clínicos randomizados (ECR) que compararam a ablação por cateter e a terapia medicamentosa em pacientes com FA e ICFEr, publicados nos últimos dez anos, disponíveis na íntegra e redigidos em português, inglês ou espanhol. A delimitação temporal foi adotada com o objetivo de contemplar evidências mais recentes, refletindo avanços nas técnicas de ablação e nas estratégias de manejo da FA nessa população.

A terapia medicamentosa para FA incluiu estratégias de controle de ritmo ou de frequência, conforme o protocolo de cada ensaio clínico incluído, mantendo-se o tratamento otimizado para insuficiência cardíaca de acordo com as recomendações das diretrizes internacionais vigentes para o manejo da doença.

Foram excluídos estudos sem relação direta com a pergunta de pesquisa, com metodologia inadequada, bem como análises secundárias, estudos *post hoc* e publicações derivadas de ECR.

### Análise e síntese de dados

Os estudos incluídos foram analisados conforme os critérios de elegibilidade estabelecidos. O desfecho primário foi a FEVE, expressa em porcentagem. Os desfechos secundários incluíram morte por todas as causas, morte cardiovascular e hospitalizações por insuficiência cardíaca. Na ausência desses desfechos, foram considerados desfechos exploratórios previamente definidos, como dimensões das câmaras cardíacas e mudança da classe funcional da NYHA.

As diferenças entre os grupos foram descritas conforme as medidas de tendência central reportadas nos estudos originais (média ou mediana), bem como as respectivas medidas de dispersão ou intervalos de confiança quando disponíveis. Devido à ausência de metanálise nesta revisão, não foi realizada conversão ou padronização dos dados, sendo os resultados apresentados conforme descritos nos estudos originais.

O risco de viés foi avaliado por dois revisores independentes por meio da ferramenta RoB 2, com resolução de divergências por consenso ou por um terceiro avaliador. As informações extraídas (identificação do artigo, intervenção, comparador, características da amostra e tempo de seguimento dos estudos) foram apresentadas em quadro de síntese, e os resultados foram descritos de forma narrativa, sem síntese quantitativa.

## Resultados

**Figura 1:**
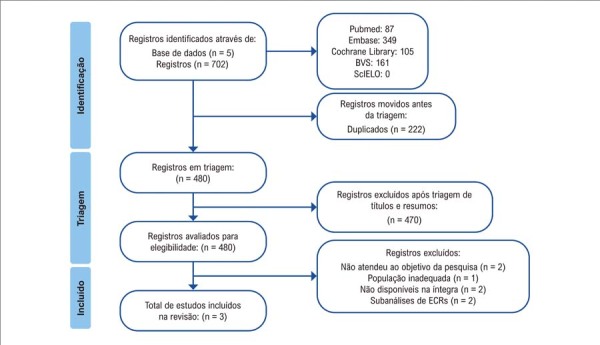
– Fluxograma PRISMA referente às etapas de seleção dos estudos. Fonte: Elaborado pelos Autores, 2025.

**Figura 2:**
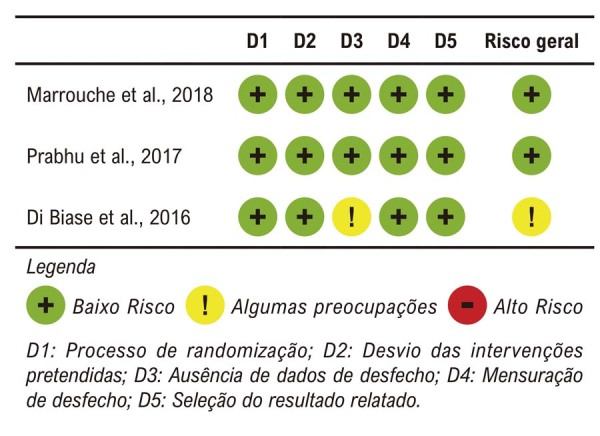
– Análise do risco de viés dos estudos incluídos. Fonte: Elaborado pelos Autores, 2025.

A busca nas bases PubMed, Embase, Cochrane Library, BVS e Scielo identificou 702 artigos (Pubmed: 87; Embase: 349; Cochrane Library: 105; BVS: 161), sem registros encontrados na Scielo. Após a remoção de 222 duplicatas, 470 artigos foram excluídos após leitura dos títulos e resumos, e 10 foram selecionados para leitura completa. Desses, 7 artigos foram retirados da análise, resultando na inclusão de três ECRs, conforme o fluxograma da Figura 1. Em relação à análise do risco de viés, nenhum dos estudos foi classificado com alto risco de viés, assim como mostrado na Figura 2. A amostra total incluiu 632 pacientes**,** predominantemente do sexo masculino, com seguimentos de 6, 24 e 60 meses. Os estudos foram codificados (E1–E3) e suas características resumidas na Tabela 1. Os principais achados deste estudo estão ilustrados na Figura Central.

**Tabela 1:** – Síntese dos aspectos gerais dos estudos

#	Título	Autor; ano e país de publicação	Intervenção	Controle	Características da amostra	Tempo de seguimento do estudo
**E1**	Catheter Ablation for Atrial Fibrillation with Heart Failure (CASTLE-AF)	Marrouche et al., 2018;^16^ Estados Unidos.	Ablação por cateter (IVP + lesões adicionais a critério do operador)	Terapia farmacológica da FA guiada por diretrizes (controle de ritmo ou frequência).	Amostra: 363 pacientes Idade (mediana): 64 anos (ambos os grupos). Sexo (mediana): Intervenção: 87% Homens; Controle: 84% Homens.	60 meses
**E2**	Catheter Ablation Versus Medical Rate Control in Atrial Fibrillation and Systolic Dysfunction: The CAMERA-MRI Study	Prabhu et al., 2017;^17^ Austrália	Ablação por cateter (IVP por ablação antral ampla + linhas adicionais no teto e parede inferior para isolamento da parede posterior)	Controle de frequência com titulação medicamentosa conforme diretrizes vigentes.	Amostra: 66 pacientes Idade (média): Intervenção: 59 anos; Controle: 62 anos. Sexo (média): Intervenção: 94% Homens; Controle: 88% Homens.	6 meses
**E3**	Ablation Versus Amiodarone for Treatment of Persistent Atrial Fibrillation in Patients With Congestive Heart Failure and an Implanted Device: Results From the AATAC Multicenter Randomized Trial	Di Biase et al., 2016;^18^ Estados Unidos	IVP por ablação antral ampla + isolamento da parede posterior do átrio esquerdo + lesões adicionais a critério do operador	Amiodarona para controle de ritmo.	Amostra: 203 pacientes Idade (média): Intervenção: 62 anos; Controle: 60 anos. Sexo (média): Intervenção: 77% Homens; Controle: 74% Homens.	24 meses

IVP: isolamento das veias pulmonares.

Fonte: Elaborado pelos autores, 2025.

### Desfechos relacionados à fração de ejeção do ventrículo esquerdo

Os três estudos avaliaram a FEVE por diferentes modalidades de imagem e tempos de seguimento. No estudo CASTLE-AF,^16^ a ablação por cateter consistiu no isolamento das veias pulmonares (IVP) com o objetivo de restabelecer o ritmo sinusal. A FEVE foi avaliada por ecocardiograma e, após 60 meses de seguimento, o grupo ablação apresentou aumento mediano de 8% na FEVE, enquanto o grupo terapia medicamentosa apresentou aumento de 0,2% (p=0,005), conforme detalhado na Tabela 2.

**Tabela 2:** – Síntese dos achados relacionados à FEVE

Estudo	Modalidade	Tempo	FEVE basal: Intervenção	FEVE basal: Controle	ΔFEVE intervenção	ΔFEVE controle	p
CASTLE-AF	Ecocardiograma	60 meses	32,5% (mediana)	31,5%. (mediana)	8,0% (2,2–19,1)	0,2% (-3,0–16,1)	0,005
CAMERA-MRI	Ressonância Magnética cardíaca	6 meses	31,8%	34,1%	18,3%	4,4%	0,0001
AATAC	Ecocardiograma	24 meses	29%	30%	8,1 ± 4	6,2 ± 5	0,02

Valores apresentados conforme reportados nos estudos originais (média, mediana ou média ± desvio-padrão). Fonte: Elaborado pelos autores.

Resultados semelhantes foram observados no estudo CAMERA-MRI.^17^ A estratégia de ablação incluiu o IVP associado ao isolamento da parede posterior do átrio esquerdo. Nesse estudo, a FEVE foi avaliada por ressonância magnética cardíaca e aumentou 18,3% no grupo ablação após seis meses, enquanto o grupo terapia medicamentosa apresentou melhora de 4,4%, com superioridade significativa do grupo ablação em relação ao controle (p<0,0001). Os valores absolutos finais da FEVE foram de 50,1% no grupo ablação e 38,4% no grupo terapia medicamentosa.

No estudo AATAC,^18^ a ablação envolveu o isolamento do antro das veias pulmonares, além do seio coronário, septo interatrial esquerdo e amplo isolamento da parede posterior do átrio esquerdo. A FEVE foi avaliada por ecocardiograma após 24 meses de seguimento, com melhora média de 8,1±4% no grupo ablação e de 6,2±5% no grupo tratado com amiodarona (p=0,02).

Vale ressaltar que tanto o estudo CASTLE-AF quanto o estudo AATAC não reportaram os valores absolutos finais de FEVE comparativos entre os grupos, apresentando apenas a variação em relação ao valor basal, conforme apresentado na Tabela 2.

### Outros desfechos analisados

No estudo CASTLE-AF,^16^ ocorreram 24 mortes no grupo ablação por cateter e 46 no grupo terapia medicamentosa (13,4% vs. 25,0%; HR 0,53; IC95% 0,32–0,86; p=0,01). A mortalidade cardiovascular também foi menor no grupo ablação (11,2% vs. 22,3%; HR 0,49; IC95% 0,29–0,84; p=0,009), assim como as hospitalizações por insuficiência cardíaca (20,7% vs. 35,9%; HR 0,56; IC95% 0,37–0,83; p=0,004).

No estudo CAMERA-MRI,^17^ os desfechos morte por todas as causas, morte cardiovascular e hospitalizações por insuficiência cardíaca não foram avaliados. Contudo, observou-se maior remodelamento reverso no grupo ablação por cateter quando comparado ao grupo terapia medicamentosa, com redução mais acentuada do volume sistólico final do ventrículo esquerdo (−24 vs. −8 ml/m^2^; p=0,007), além de redução do volume do átrio esquerdo em 12 ml/m^2^ e melhora significativa da classe funcional da NYHA (p<0,0001).

No estudo AATAC,^18^ embora não tenham sido avaliados desfechos específicos como hospitalizações por insuficiência cardíaca ou morte cardiovascular, o grupo ablação apresentou menor taxa de hospitalizações não planejadas (31% vs. 57%; RR 0,55; IC95% 0,39–0,76; p<0,001) e menor mortalidade por todas as causas (8% vs. 18%; RR 0,44; IC95% 0,20–0,96; p=0,037).

## Discussão

Nos estudos analisados nesta revisão, a ablação por cateter foi associada à melhora da FEVE em pacientes com FA e ICFEr, em comparação à terapia medicamentosa. O aumento da FEVE foi observado ao longo do seguimento dos estudos incluídos. Além disso, essa melhora esteve associada, em alguns dos estudos, à redução da mortalidade por todas as causas, mortalidade cardiovascular e hospitalizações por IC, sugerindo impacto positivo da ablação no prognóstico clínico desses pacientes.

Dados observacionais derivados dos ensaios CAMTAF e ARC demonstraram aumento médio de 8,4% da FEVE no grupo submetido à ablação por cateter, em contraste com redução no grupo tratado clinicamente, além de melhores escores de qualidade de vida pelo Minnesota Living With Heart Failure Questionnaire (MLWHFQ),^19^ reforçando esses achados.

O aumento da FEVE observado nos três ensaios sugere que, embora ambos os grupos tenham apresentado melhora, a ablação permitiu alcançar limiares clinicamente mais seguros, com redução da disfunção ventricular e possível impacto positivo na qualidade de vida. Tais achados são corroborados por estudos que demonstraram que a recuperação da função ventricular tem sido associada a melhores escores no MLWHFQ, além de menores taxas de mortalidade por todas as causas e de hospitalizações por insuficiência cardíaca no grupo submetido à ablação por cateter.^20,21^

Ademais, as diferenças nas estratégias de ablação podem influenciar a magnitude da recuperação ventricular. No estudo CASTLE-AF,^16^ a abordagem centrada no IVP foi associada a aumento da FEVE e à redução de mortalidade e hospitalizações, em concordância com estudos que demonstraram boa eficácia da ablação baseada exclusivamente no IVP em pacientes com FA paroxística.^22,23^

Em contrapartida, o estudo CAMERA-MRI^17^empregou IVP associado ao isolamento da parede posterior do átrio esquerdo, resultando em aumento médio de 18,5% da FEVE em seis meses e normalização em mais da metade dos pacientes. Já o estudo AATAC^18^ utilizou estratégia intermediária, combinando IVP com linhas adicionais, alcançando melhora sustentada da FEVE e menor número de hospitalizações não planejadas em seguimento prolongado. Esses dados estão em concordância com a literatura que sugere a associação de estratégias de ablação mais amplas a maiores benefícios estruturais e funcionais, sobretudo em pacientes com FA persistente e disfunção ventricular significativa, em que os mecanismos perpetuadores da arritmia são mais difusos.^24,25^

De forma complementar, os estudos CASTLE-AF^16^ e AATAC^18^ demonstraram benefícios clínicos da estratégia intervencionista, especialmente na redução de hospitalizações e da mortalidade. No estudo AATAC,^18^ a menor taxa de hospitalizações não planejadas e a redução da mortalidade por todas as causas sugerem que um controle mais efetivo do ritmo melhora o prognóstico dessa população. Nesse contexto, meta-análises recentes também têm descrito superioridade da ablação em relação à terapia medicamentosa isolada, destacando a intervenção por cateter como potencial modificadora de desfechos clínicos desfavoráveis em pacientes com alto risco cardiovascular.^26,27^

A redução do volume sistólico final do ventrículo esquerdo e do volume do átrio esquerdo observada no grupo submetido à ablação sugere possível impacto dessa estratégia na remodelação cardíaca reversa, aspecto fundamental para a melhora do prognóstico nesses pacientes.^28,29^ Além disso, a melhora da classe funcional pela NYHA nesse grupo sugere a associação entre as alterações estruturais e a evolução clínica mais favorável. Esses resultados indicam que a ablação pode exercer um papel relevante na evolução da doença, ao favorecer o remodelamento cardíaco reverso e a melhora da sintomatologia nessa população.^30,31^

Dentre as limitações desta revisão, destaca-se a ausência de metanálise, o que impede a padronização quantitativa dos resultados. Soma-se a isso a quantidade limitada de artigos incluídos, o que dificulta a generalização dos achados e a consolidação de indicações terapêuticas para a prática clínica. Novos ECR e análises quantitativas são necessários para confirmar a magnitude desses efeitos e melhor definir o papel da ablação por cateter nessa população.

## Conclusão

A análise narrativa dos ensaios clínicos incluídos sugere que a ablação está associada à melhora da fração de ejeção do ventrículo esquerdo quando comparada à terapia medicamentosa em pacientes com FA e ICFEr. Alguns estudos relataram ainda melhora da qualidade de vida e das condições clínicas dos pacientes submetidos à ablação. Além disso, no que se refere especificamente à mortalidade cardiovascular e às hospitalizações por insuficiência cardíaca, o estudo CASTLE-AF observou redução de ambos os desfechos nesse grupo de pacientes. Entretanto, como esta revisão se baseia em uma síntese narrativa dos estudos disponíveis, não foi realizada uma análise quantitativa dos desfechos. Embora os resultados se mostrem promissores quanto à aplicação da técnica de ablação nessa população, estudos adicionais, com amostras maiores e metodologias comparáveis, são necessários para confirmar e ampliar a generalização desses achados.
