# Supplementary material for: Impacto da Ablação por Cateter na FEVE em Pacientes com Fibrilação Atrial e Insuficiência Cardíaca com Fração de Ejeção Reduzida: Uma Revisão Sistemática
Source: Arq Bras Cardiol. 2026 Jun 26;123(6):e20260117. [Article in Portuguese] doi: 10.36660/abc.20260117 (PMC13399039; doi:10.36660/abc.20260117)
Supplement: Translation

# Translation: Impact of Catheter Ablation on LVEF in Patients with Atrial Fibrillation and Heart Failure with Reduced Ejection Fraction: A Systematic Review

**DOI:** 10.36660/abc.20260117i

**Authors:** Antonio Maria Zacarias Araújo Monteiro, Maria Giovanna da Cruz Tocantins, Jéssica Batista Souza, Renan de Jesus da Silva Albuquerque, João Vitor Ferreira de Oliveira, Roni Oliveira Pinheiro, Ster Marques de Lima Silva

**Affiliations:** 1 Brazil Centro Universitário do Estado do Pará, Belém, PA – Brazil; 2 Brazil Universidade do Estado do Pará, Belém, PA – Brazil; 3 Brazil Universidade Federal do Pará, Belém, PA – Brazil

**Keywords:** Heart Failure, Atrial Fibrillation, Catheter Ablation, Drug Therapy

## Abstract

Atrial fibrillation (AF) is a condition that frequently affects patients with heart failure with reduced ejection fraction (HFrEF) and is associated with worsening left ventricular ejection fraction (LVEF) and unfavorable clinical outcomes. Catheter ablation has been proposed as a strategy to restore sinus rhythm; however, its effects on LVEF remain heterogeneous in the literature. To evaluate the impact of catheter ablation, compared with medical therapy, on LVEF in patients with AF and HFrEF. A systematic review of randomized clinical trials was conducted in accordance with the PRISMA guidelines. The primary outcome was improvement in LVEF, while secondary outcomes included cardiovascular mortality, all-cause mortality, and hospitalizations due to heart failure. Other exploratory outcomes were also considered. The included randomized clinical trials demonstrated improvement in LVEF among patients undergoing catheter ablation compared with those receiving medical therapy. In the CAMERA-MRI study, an absolute increase of 18.3% in LVEF was observed after six months. In the CASTLE-AF trial, catheter ablation was also associated with a lower incidence of cardiovascular mortality and heart failure hospitalizations. In the absence of a meta-analysis, no quantitative synthesis of the effects was performed. The available evidence from individual randomized trials suggests that catheter ablation may be associated with improvement in LVEF and more favorable clinical outcomes in patients with AF and HFrEF. However, given the lack of quantitative synthesis, these findings should be interpreted with caution.

## Introduction

Heart failure (HF) is a syndrome resulting from abnormalities in ventricular filling and/or ejection and is associated with high mortality and a significant burden of clinical complications, including dyspnea, exercise intolerance, and lower limb edema.^1^ Traditionally, HF is classified according to left ventricular ejection fraction (LVEF) into three categories: preserved, mildly reduced (or intermediate), and reduced (HFrEF, LVEF ≤ 40%),^1^ the latter accounting for approximately 50% of hospitalizations due to HF.^2^

Atrial fibrillation (AF) is common in this setting, affecting approximately one-third of patients with HFrEF.^3^ The coexistence of these conditions is associated with a worse prognosis, with an increased risk of stroke, hospitalizations, and mortality.^4,5^

The therapeutic management of AF in patients with HFrEF has traditionally focused on rate control or restoration of sinus rhythm. Beta-blockers represent the first-line therapy for heart rate control.^6^ However, their use remains controversial, as some studies have not demonstrated significant reductions in mortality.^7,8^ Rhythm control with antiarrhythmic drugs, particularly amiodarone, represents another important therapeutic option; however, its use is limited by the occurrence of relevant adverse events in this population.^9,10^

Catheter ablation has therefore emerged as a therapeutic alternative to antiarrhythmic drugs and has been associated with improvements in LVEF, as well as reductions in clinically relevant outcomes such as mortality and heart failure hospitalizations.^11,12^ However, there is a scarcity of studies specifically evaluating the effects of ablation in patients with HFrEF, particularly with regard to LVEF outcomes. Furthermore, considerable heterogeneity exists among studies regarding inclusion criteria, ablation protocols, medical therapies, and the definition of procedural success, which limits the generalizability and interpretation of the results.^13,14^

Therefore, this review aims to evaluate the efficacy of catheter ablation compared with medical therapy in improving LVEF in patients with AF and HFrEF, considering the heterogeneity of the available studies.

## Methods

This study was conducted as a systematic review in accordance with the Preferred Reporting Items for Systematic Reviews and Meta-Analyses (PRISMA) guidelines,^15^ with the protocol previously registered in PROSPERO (CRD420251044229).

### Literature Search and Study Selection

The research question was formulated based on the PICO strategy, considering patients with AF and heart failure with reduced ejection fraction (HFrEF), comparing catheter ablation with medical therapy regarding improvement in LVEF.

The literature search was conducted in March 2025 in the PubMed, BVS, Cochrane Library, SciELO, and EMBASE databases using the descriptors *“Atrial Fibrillation,” “Heart Failure,” “Catheter Ablation,”* and *“Drug Therapy”* (DeCS/MeSH), combined with the Boolean operator AND. The identified studies were imported into the Rayyan^®^ platform and independently screened by two reviewers.

### Inclusion and exclusion criteria

Randomized controlled trials (RCTs) comparing catheter ablation and medical therapy in patients with AF and HFrEF were included. Eligible studies were those published within the last ten years, available in full text, and written in Portuguese, English, or Spanish. This temporal restriction was adopted to include more recent evidence, reflecting advances in ablation techniques and in the management strategies for AF in this population.

Medical therapy for AF included rhythm-control or rate-control strategies, according to the protocol of each included clinical trial, while maintaining optimized heart failure treatment in accordance with current international guideline recommendations for the management of the disease.

Studies not directly related to the research question, those with inadequate methodology, as well as secondary analyses, post hoc analyses, and publications derived from randomized clinical trials were excluded.

### Data analysis and synthesis

The included studies were analyzed according to the established eligibility criteria. The primary outcome was LVEF, expressed as a percentage. Secondary outcomes included all-cause mortality, cardiovascular mortality, and hospitalizations due to heart failure. In the absence of these outcomes, previously defined exploratory outcomes were considered, such as cardiac chamber dimensions and changes in New York Heart Association (NYHA) functional class.

Differences between groups were described according to the measures of central tendency reported in the original studies (mean or median), as well as the corresponding measures of dispersion or confidence intervals, when available. Due to the absence of a meta-analysis in this review, no conversion or standardization of data was performed, and the results were presented as reported in the original studies.

The risk of bias was independently assessed by two reviewers using the RoB 2 tool, with disagreements resolved by consensus or by a third reviewer. Extracted information (article identification, intervention, comparator, sample characteristics, and study follow-up time) was presented in a summary table, and the results were described narratively, without quantitative synthesis.

## Results

The search of PubMed, Embase, Cochrane Library, and BVS databases identified 702 records (PubMed: 87; Embase: 349; Cochrane Library: 105; BVS: 161), with no articles retrieved from SciELO. After removal of 222 duplicates, 470 records were excluded based on title and abstract screening, and 10 articles were selected for full-text review. Of these, seven were excluded, resulting in the inclusion of three randomized controlled trials (RCTs), as illustrated in the flow diagram (Figure 1). Regarding the risk of bias analysis, none of the studies showed a high risk of bias, as illustrated in Figure 2.

**Figure 1:** – PRISMA flow diagram of the study selection process. Source: Prepared by the authors, 2025.

**Figure 2:** – Risk of bias analysis of selected articles. Source: Prepared by the authors, 2025.

The final sample comprised 632 patients, predominantly male, with follow-up periods of 6, 24, and 60 months. The studies were coded as E1–E3, and their main characteristics are summarized in Table 1. The main findings of the study are illustrated in the Central Illustration.

**Table 1:** – Summary of the general characteristics of the included studies

#	Title	Author(s), year, and country	Intervention	Control	Sample characteristics and primary outcome	Study follow-up duration
**E1**	Catheter Ablation for Atrial Fibrillation with Heart Failure (CASTLE-AF)	Marrouche et al., 2018;^16^ United States of America	Catheter ablation (pulmonary vein isolation [PVI] + additional lesions at the operator’s discretion)	Guideline-directed pharmacological therapy for AF (rhythm-control or rate-control strategy)	Sample: 363 patients Age (median): 64 years (both groups). Sex (median): Intervention: 87% male; Control: 84% male.	60 months
**E2**	Catheter Ablation Versus Medical Rate Control in Atrial Fibrillation and Systolic Dysfunction: The CAMERA-MRI Study	Prabhu et al., 2017;^17^ Australia.	Catheter ablation (PVI by wide antral ablation + additional roof and inferior lines for posterior wall isolation)	Rate control with medication titration according to current guideline recommendations.	Sample: 66 patients Age (mean): Intervention: 59 years; Control: 62 years. Sex (mean): Intervention: 94% male; Control: 88% male.	6 months
**E3**	Ablation Versus Amiodarone for Treatment of Persistent Atrial Fibrillation in Patients With Congestive Heart Failure and an Implanted Device: Results From the AATAC Multicenter Randomized Trial	Di Biase et al., 2016;^18^ United United States of America	PVI by wide antral ablation + left atrial posterior wall isolation + additional lesions at the operator’s discretion	Amiodarone for rhythm control.	Sample: 203 patients Age (mean): Intervention: 62 years; MT: 60 years. Sex (mean): Intervention: 77% male; Control: 74% male.	24 months

Source: Prepared by the authors, 2025.

### LVEF-related outcomes

The three studies assessed LVEF using different imaging modalities and follow-up periods. In the CASTLE-AF study,^16^ catheter ablation consisted of pulmonary vein isolation (PVI) with the aim of restoring sinus rhythm. LVEF was evaluated by echocardiography, and after 60 months of follow-up, the ablation group showed a median increase of 8% in LVEF, whereas the medical therapy group showed an increase of 0.2% (p = 0.005), as detailed in Table 2.

**Table 2:** – Summary of LVEF-Related Findings

Study	Imaging Modality	Follow-up	Baseline LVEF: Intervention	Baseline LVEF: Control	ΔLVEF Intervention	ΔLVEF Control	p-value
CASTLE-AF	Echocardiography	60 months	32.5% (median)	31.5% (median)	8.0% (2.2–19.1)	0.2% (-3.0–16.1)	0.005
CAMERA-MRI	Cardiac Magnetic Resonance	6 months	31,8%	34,1%	18.3%	4.4%	0.0001
AATAC	Echocardiography	24 months	29%	30%	8.1 ± 4	6.2 ± 5	0.02

Values are presented as reported in the original studies (mean, median, or mean ± standard deviation). Source: Prepared by the authors.

Similar findings were observed in the CAMERA-MRI study.^17^ The ablation strategy included pulmonary vein isolation (PVI) combined with posterior wall isolation of the left atrium. In this study, LVEF was assessed by cardiac magnetic resonance imaging and increased by 18.3% in the ablation group after six months, whereas the medical therapy group showed an improvement of 4.4%, with significant superiority of the ablation group compared with the control group (p < 0.0001). The final absolute LVEF values were 50.1% in the ablation group and 38.4% in the medical therapy group.

In the AATAC study,^18^ the ablation strategy involved pulmonary vein antral isolation, in addition to ablation of the coronary sinus, the left interatrial septum, and extensive isolation of the posterior wall of the left atrium. LVEF was assessed by echocardiography after 24 months of follow-up, showing a mean improvement of 8.1 ± 4% in the ablation group and 6.2 ± 5% in the amiodarone-treated group (p = 0.02).

It is noteworthy that both the CASTLE-AF and AATAC^16-18^ studies did not report the final absolute LVEF values for comparison between groups, presenting only the change relative to baseline, as shown in Table 2.

### Other analyzed outcomes

In the CASTLE-AF study,^16^ 24 deaths occurred in the catheter ablation group and 46 in the medical therapy group (13.4% vs. 25.0%; HR 0.53; 95% CI 0.32–0.86; p=0.01). Cardiovascular mortality was also lower in the ablation group (11.2% vs. 22.3%; HR 0.49; 95% CI 0.29–0.84; p=0.009), as were hospitalizations for heart failure (20.7% vs. 35.9%; HR 0.56; 95% CI 0.37–0.83; p=0.004).

In the CAMERA-MRI study,^17^ the outcomes of all-cause mortality, cardiovascular mortality, and hospitalizations for heart failure were not evaluated. However, greater reverse remodeling was observed in the catheter ablation group compared with the medical therapy group, with a more pronounced reduction in left ventricular end-systolic volume (−24 vs. −8 mL/m^2^; p=0.007). Additionally, a reduction in left atrial volume of 12 mL/m^2^ and a significant improvement in New York Heart Association (NYHA) functional class were observed (p<0.0001).

In the AATAC study,^18^ although specific outcomes such as hospitalizations for heart failure or cardiovascular mortality were not evaluated, the ablation group showed a lower rate of unplanned hospitalizations (31% vs. 57%; RR 0.55; 95% CI 0.39–0.76; p<0.001) and lower all-cause mortality (8% vs. 18%; RR 0.44; 95% CI 0.20–0.96; p=0.037).

## Discussion

In the studies analyzed in this review, catheter ablation was associated with an improvement in LVEF in patients with AF and HFrEF, compared with medical therapy. The increase in LVEF was observed throughout the follow-up period of the included studies. Furthermore, in some of the studies, this improvement was associated with reductions in all-cause mortality, cardiovascular mortality, and hospitalizations for heart failure, suggesting a positive impact of ablation on the clinical prognosis of these patients.

Observational data derived from the CAMTAF and ARC trials demonstrated a mean increase of 8.4% in LVEF in the group undergoing catheter ablation, in contrast to a reduction in the group treated clinically. Additionally, better quality-of-life scores were observed according to the Minnesota Living With Heart Failure Questionnaire (MLWHFQ),^19^ further supporting these findings.

The increase in LVEF observed across the three trials suggests that, although both groups showed improvement, catheter ablation enabled the achievement of clinically safer thresholds, with a reduction of ventricular dysfunction and a potential positive impact on quality of life. These findings are supported by studies demonstrating that recovery of ventricular function has been associated with better scores on the Minnesota Living with Heart Failure Questionnaire, as well as lower rates of all-cause mortality and hospitalizations for heart failure in the catheter ablation group.^20,21^

Furthermore, differences in ablation strategies may influence the magnitude of ventricular recovery. In the CASTLE-AF^16^ study, a pulmonary vein isolation (PVI)-focused approach was associated with increased LVEF and reduced mortality and hospitalizations, in agreement with studies demonstrating good efficacy of ablation based exclusively on PVI in patients with paroxysmal AF.^22,23^

In contrast, the CAMERA-MRI^17^ study employed PVI combined with posterior left atrial wall isolation, resulting in a mean LVEF increase of 18.5% at six months and normalization in more than half of the patients. The AATAC^18^study used an intermediate strategy, combining PVI with additional linear lesions, achieving sustained LVEF improvement and fewer unplanned hospitalizations during long-term follow-up. These findings are consistent with the literature suggesting that more extensive ablation strategies are associated with greater structural and functional benefits, particularly in patients with persistent AF and significant ventricular dysfunction, in whom arrhythmia-perpetuating mechanisms are more diffuse.^24,25^

Complementarily, the CASTLE-AF^16^ and AATAC^18^studies demonstrated clinical benefits of the interventional strategy, particularly in reducing hospitalizations and mortality. In the AATAC^18^study, the lower rate of unplanned hospitalizations and reduced all-cause mortality suggest that more effective rhythm control improves the prognosis in this population. In this context, recent meta-analyses have also reported the superiority of ablation over medical therapy alone, highlighting catheter-based intervention as a potential modifier of adverse clinical outcomes in patients at high cardiovascular risk.^26,27^

The reduction in left ventricular end-systolic volume and left atrial volume observed in the ablation group suggests a potential impact of this strategy on reverse cardiac remodeling, a key factor for improved prognosis in these patients.^28,29^ Furthermore, the improvement in NYHA functional class in this group indicates an association between structural changes and more favorable clinical outcomes. These results suggest that ablation may play a relevant role in disease progression by promoting reverse cardiac remodeling and symptomatic improvement in this population.^30,31^

Among the limitations of this review, the absence of a meta-analysis stands out, preventing quantitative standardization of the results. In addition, the limited number of included studies hinders the generalization of the findings and the consolidation of therapeutic recommendations for clinical practice. Further randomized clinical trials and quantitative analyses are needed to confirm the magnitude of these effects and better define the role of catheter ablation in this population.

## Conclusion

The narrative analysis of the included clinical trials suggests that ablation is associated with improved LVEF compared to medical therapy in patients with AF and HFrEF. Some studies also reported improvements in quality of life and clinical status among patients undergoing ablation. Furthermore, with regard specifically to cardiovascular mortality and heart failure-related hospitalizations, the CASTLE-AF study observed reductions in both outcomes in this patient group. However, as this review is based on a narrative synthesis of the available studies, no quantitative analysis of outcomes was performed. Although the results appear promising regarding the application of the ablation technique in this population, further studies with larger sample sizes and comparable methodologies are needed to confirm and broaden the generalizability of these findings.
